# Improving the Translational Medicine Process: Moving Patients From “End-Users” to “Engaged Collaborators”

**DOI:** 10.3389/fmed.2019.00110

**Published:** 2019-05-21

**Authors:** Manuela Battaglia, Pat Furlong, Nico Martinus Wulffraat, Felicitas Bellutti Enders

**Affiliations:** ^1^Diabetes Research Institute, IRCCS San Raffaele Scientific Institute, Milan, Italy; ^2^Parent Project Muscular Dystrophy, Hackensack, NJ, United States; ^3^Department of Pediatric Immunology, Wilhelmina Children's Hospital, University Medical Centre Utrecht, Utrecht, Netherlands; ^4^Allergy Unit, Department of Dermatology, University Hospital, Basel, Switzerland

**Keywords:** translational medicine, patient-centric approaches, shared decision medicine, the innovation journey, patient advocacy

## Abstract

Translational medicine works through the definition of unmet medical needs, their understanding and final resolution. In this complex and multi-disciplinary process patients have always been regarded as “end-users” or no more than “data provider.” Considering that the translational practice is nowadays highly inefficient (i.e., large intellectual and economical resources are wasted with limited impact on people health) here we propose to reverse the process: start from patients, engage them, and keep them at the center. A new partnership needs to be formed between the patients and the health care professionals, as well as the treating physicians, to make the most out of the current “health resources.” New patient-centric approaches are emerging but they remain isolated phenomena often difficult to implement. Here—with this perspective—we aim at thinking differently and learning from new experiences. We will provide some successful examples of change, and we will discuss new approaches to create a radical change in the way translational medicine is managed and how this would significantly impact people health and health care systems.

## Introduction

The European Society for Translational Medicine has defined translational medicine as an interdisciplinary branch of the biomedical field supported by three main pillars: benchside, bedside, and community. The goal of translational medicine is to combine disciplines, resources, expertise, and techniques within these pillars to translate efficiently and effectively scientific research findings relevant to human diseases into knowledge that is beneficial for patients via new drugs, devices, or treatment options (1). Accordingly, translational medicine is a highly interdisciplinary field and includes academia, industry, and regulatory institutions. However, patients (who are the direct beneficiaries of translational medicine) are often excluded by this complex process.

In this perspective paper, we will discuss the impasse of translational medicine, the role that patients should have in this process (with concrete examples of success) and the future directions with the aim at fostering a science that really impacts on patients' life.

## The Impasse of Translational Medicine

Translational medicine is a process fundamental for the society as it aims at developing new interventions beneficial to the patients. However, translational medicine is at a historic moment of crisis. The process is becoming unsustainable in spite of enormous technological advances, since the technological explosion has not been accompanied by a reinforcement of quality in experimental designs, especially in the discovery phases. However, there is no clear path neither for clinicians nor for scientists regarding the process of how a discovery leads to an approved drug. The high level of failure of clinical trials in Phase II/III swallows up economic resources, generates exhaustion among researchers and clinicians and, most importantly, induces frustration among patients who see their hopes for a new drug to treat their disease, disappear (2). The high failure of clinical trials can be due to the inadequate study design, incorporating endpoints that provide limited or misleading information regarding the efficacy of the test agent, to the limited reproducibility of data, or also due to the high variability between tested subjects regarding their genetic background or the heterogeneity of their disease and also their comorbidities (3). Although it is now clear that even the failure of well-designed studies benefits both researchers and healthcare systems by, for example, generating evidence about disease theories and demonstrating the limits of proven drugs (4).

The Food and Drug Administration (FDA) publishes every year a list of all the drugs released on the market ^1^. Backtracking the initial publication on the mechanism or molecule leading to drug development shows—for the drugs released between January and September 2018—a median interval of 10 years (range: 5–37 years) before the drug is reaching the patient. This is becoming unsustainable as it creates tremendous social distress. At a time of global financial crisis, citizens perceive that vital resources are not being used efficiently and scientists fear to enter in a career path that is uncertain and not properly rewarded.

Thus, there is a great need to reconsider the translational medicine process and we do believe that moving the patients from “end-users” to “engaged collaborators” would transform them into agents of change. The standard business model is indeed to speak to the consumer. Apple for instance understands its consumer: it must first identify the customer, talk about the product and ask if the intended consumer would value the product. Is it any wonder why Apple is the first billion-dollar company? They know their consumer! In translational medicine, this concept is ignored. Patients are the ultimate users of health technologies and they can advocate and promote models for patient involvement among other stakeholders. Nothing will facilitate the dialogue among scientists, clinicians, and society more effectively than the creation of a pathway, constructed together, and bound by a common objective. This should lead to improved translational medicine efficiency and reduced waste of resources and energies.

## Current Advances

The doctor-patient relationship in western countries has significantly evolved over the years. Prior to the last two decades the relationship followed a paternalistic model, where the patient sought help and the doctors used their skills to choose the necessary interventions or treatments to restore or improve patients' health. Decisions of the doctors were silently complied by the patient (5). The social system has been challenged over the last 20 years: society has changed (being now multicultural), access to information is broader (social), media allow easier contact between patients and thus facilitated creation of patient's organization. Therefore, critics have emerged, demanding a more active, autonomous and thus centered role for the patient who advocates greater control, reduced physician dominance, and more mutual participation.

This has led to the idea of the Shared Decision Medicine (SDM), which is a process promoted by the Institute of Medicine (IOM) as part of the strategy to improve the quality of health care in the United States. The IOM recommended that healthcare should be customized based on the patient's needs and values, the patient should be given adequate knowledge and control to make decisions that affect his/her health, patients and healthcare providers should communicate and share information, and patients should receive information that allows them to make informed decisions. To this end, SDM is the joint involvement of patients and healthcare providers in making healthcare decisions that are informed by the best available evidence in regards to possible options, potential benefits and harms, and that consider patient preferences and values. SDM ensures patients get no more and no less of the care they need and want (6, 7). However, despite attention to principles and competences, there remains a lack of clear guidance about how to accomplish SDM in routine practice. Studies have not yet addressed the question about the impact on professionals. There might be the need to coach patients to be able to assess the value, risks, benefits, and burden of interventions. For organizations, a consistent shared decision-making might change patient experience evaluations and lead to a “satisfied patient” and fewer complaints or even legal issues. Clear outcome measurements of shared decision-making are needed as they would provide a more substantive evidence base to guide implementation (6).

Another, more recent, approach to bring the patients closer to the science that could impact their life is the “plan S.” Research funders from France, the United Kingdom, the Netherlands, and eight other European nations have unveiled a radical open-access initiative; they will mandate that, from 2020, the scientists they fund must make resulting papers free to read immediately on publication. The scientific papers would have a liberal publishing license that would allow anyone to download, read it or otherwise reuse the work leading to a science no more locked behind paywalls and freely available for everybody (8).

Increasingly, funding opportunities for translational biomedical research require studies to engage community partners, patients, or other stakeholders in the research process to address their concerns. However, there is little evidence on strategies to prepare teams of academic and community partners to collaborate on grants. A well-planned and feasible educational program designed to help community organizations and academic institutions to build infrastructure for collaborative research projects using a partnered approach is needed and some institutions are already investing in this important activities (9, 10).

Industry is today also very open to view patients as close collaborators and aims at connecting with them throughout the innovation journey, starting with validation of new concepts to the design of patient-centric trials. The customer journey is a term from marketing, describing the 5 cycles, which a client passes through before he decides to buy a product, or in medical terms, before he decides for one or the other therapy. Five phases mark this journey: awareness, favorability, consideration, intent to purchase (or in medical terms, intent to treat), conversion (decision to treat). For most pharma companies, this represents a major shift in thinking. It requires putting not the product but the customer at the center of the launch, and addressing customers' emotional and behavioral needs as well as their clinical ones.

There are also tangible examples of success on how to move the patients at the center of the translational medicine process. Here we report two specific cases.

### The Remarkable Story of a Mother and the Parent Project Muscolar Dystrophy

**Figure 1:**
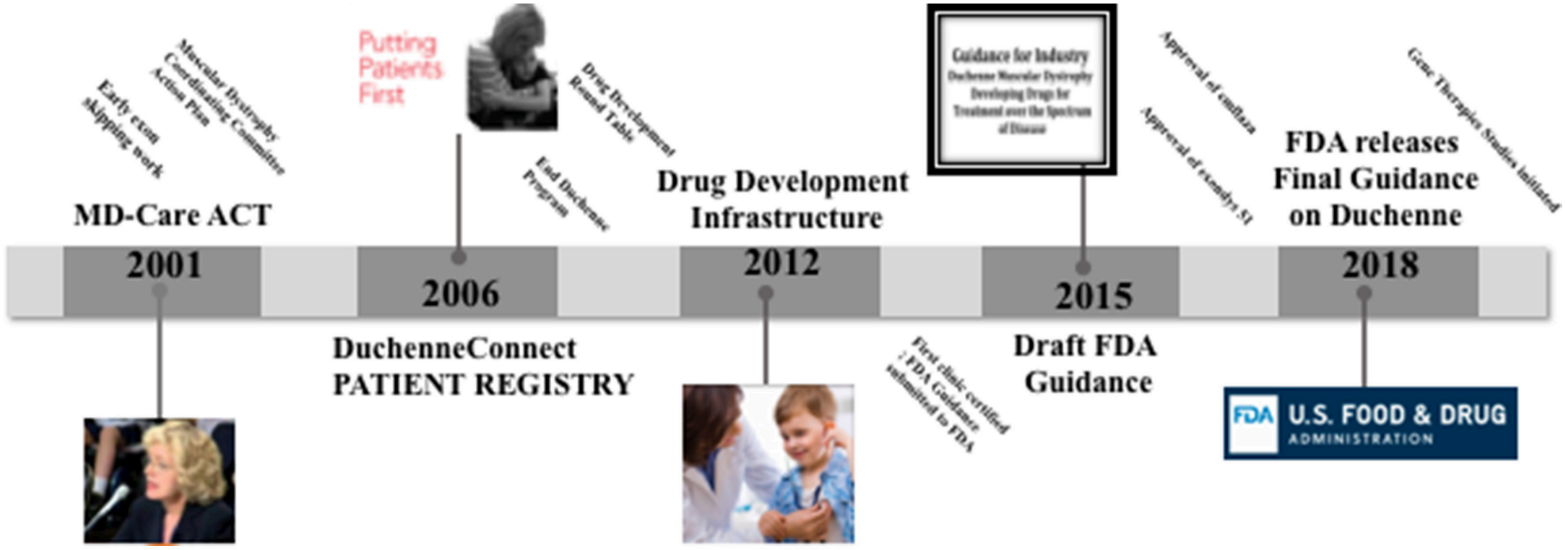
Time line of the parent project muscular dystrophy (PPMD) contributions to Duchenne care and treatment.

When doctors diagnosed her two sons, Christopher and Patrick, with Duchenne muscular dystrophy (DMD) in 1984, Pat Furlong didn't accept the therapeutic nihilism, the fatalistic message from their doctor “there is no hope and little help.” DMD is the most common, lethal genetic disease diagnosed in childhood; it is an aggressive and ultimately fatal muscle wasting disease that primarily affects boys and it results in a progressive loss of muscle strength. Individuals with DMD lose ambulation in the early teens, require ventilation in the mid-teens and die before reaching the 3rd decade. Families who receive the diagnosis are in a race against time. They await new knowledge and scientific breakthroughs, possibilities to slow the degeneration. As of today, steroids are used to slow the decline, but there are no cures for DMD. In 1994, together with other parents, Pat Furlong founded Parent Project Muscular Dystrophy (PPMD) to change the course of the disease and ultimately end DMD. PPMD is today the largest most comprehensive non-profit organization in the United States focused on finding a cure for DMD ^2^. In her quest for a cure, she first realized that there simply wasn't enough research into the disease and too many questions being left unanswered. Her first efforts focused on small investments in academic research and leveraging those investments. Due to the rarity of the disease (1 in 4600−5600 boys) and hence lack of potential profits there had been little interest at the onset from major pharmaceutical companies. Early in the fight, PPMD realized that the greatest source of advancement in basic science surrounding DMD would be through an investment by the National Institutes of Health (NIH) and related agencies. In 2000, the Duchenne community, through PPMD, employed a firm to lobby on their behalf in Washington DC and scored major legislative success with the introduction of legislation, intended to require government agencies such as NIH to significantly increase its investment in and coordination of research into the muscular dystrophy's. That same year, at the insistence of PPMD, NIH held a scientific workshop on Duchenne, bringing in scientists from all over the world to advance the cause. This was a workshop of major significance in which attending scientists' and researchers came to the realization that with the knowledge of the genetic basis of the disease and through multidisciplinary collaboration, something could be done to improve the quality of life and extend the lifespan of boys with DMD. On the tails of the earlier success with NIH, PPMD continued its Washington DC advocacy agenda and achieved another stirring victory. In December 2001, the Muscular Dystrophy CARE ACT was signed into law. This legislation dramatically increased NIHs investment in Muscular dystrophy research (from ~$17 millions to over $750 millions), including the funding of six Centers of Excellence. All of that, in addition to the earlier orphan drug act of 1983 incentivizing companies to invest in rare disease research, resulted in significant breakthroughs and new knowledge to fully characterize the pathology of DMD and to encourage industry interest in targeting relevant pathways. Today there are more than 40 ongoing clinical trials, whereas in 1999 there was 1 trial. Additionally, today there are more than 45 pharmaceutical companies investing in DMD. Current market estimates an 8-Billion-dollar investment in drug development. PPMD is currently working with FDA to develop a Master Protocol to enable access to trials across the Duchenne community, potentially leading to combination therapies and reach the highest priority of families (Figure 1).

Patient advocacy has come of age. Foundations focused on a specific disease provide substantial investments in research, organize the patient community, collect data to better understand disease progression, support the development of biobanks, inform regulatory interactions and assist patients navigate the healthcare environment. Advocacy efforts lead the ecosystem of research, therapy development, access and reimbursement. Her sons lost their battle with DMD in their teenage years, but Pat Furlong continues to fight—in their honor and for all the community to this day.

### The Development of a Patient Council Within a Clinical Department

Another, yet different, example of success comes from the Department of Pediatric Rheumatology at the Wilhelmina Children's University Hospital in Utrecht (The Netherland) focused on the study and cure of Juvenile idiopathic arthritis (JIA). JIA is the most common chronic rheumatic disorder in children and is a major cause of short-term and long-term disability. JIA is defined as having an inflamed joint before the age of 16 without a clear cause that persists for more than 6 weeks; it is a chronic disorder, which if neglected, can lead to serious complications.

In developing a network for biological research for patients with Childhood Arthritis doctors and scientists at the Wilhelmina Center of Excellence strongly think that input from and collaboration with patients and patient organizations is crucial. Patients, their parents, doctors and researchers all share the same common goal, namely that progress in basic science is translated in real tangible products for patients with childhood arthritis. In 2013 a patient council was formed in this Department. Together with professionals the JIA patient council explore research priority setting by reviewing the research topics, safety and efficacy of immunizations, as well as stopping medications. In addition to this, a jointly written application was obtained for a project with focus groups for patients that was also led by a parent. The patient council selected a topic which was the most frequent concern expressed by patients: the uncertainty patients feel due to the impact of the unpredictable course of their disease (pain, relapses) in their activities of daily life (activities at school for younger children and later work, sports and social contacts). Focus groups further analyzed the effects of the unpredictable course of the disease. Information was written for websites and two youtube movies were made. The group made of Dutch organizations of patients, parents and clinicians will collaboratively develop a research agenda for JIA, following the James Lind Alliance (JLA) methodology ^3^. The JLA is a non-profit making initiative established in 2004 and it brings patients, caregivers and clinicians together in Priority Setting Partnerships (PSPs) to identify and prioritize the top 10 uncertainties, or unanswered questions, about the effects of treatments. The aim of this is to make sure that health research funders are aware of the issues that matter most to patients and clinicians. In this process the input from clinicians, patients and their caregivers will be equally valued. Additionally, focus groups will be organized to involve young people with JIA. The involvement of all contributors will be monitored and evaluated. In this manner, the project will contribute to the growing body of literature on how to involve young people in agenda setting in a meaningful way.

This approach, despite still at its infancy, will inform researchers and research funders about the most important research questions for JIA and this will hopefully lead research agenda for research that really matters (11).

## Current Obstacles and Future Directions

The examples provided show how patients and their care givers can be the catalysts of a change that is highly needed in translational medicine but they remain, as per today, sporadic cases led by unique human beings or by particularly inspired institutions. Many obstacles remain. Qualitative research showed that the involvement of patients and caregivers is challenging: real co-design does not happen by itself (12). First, specific educational programs are needed to improve the process of shared decision-making, for both partners, the patient and the physician. These programs are missing and importantly clinicians are often limited in their time-management. Educate and engage patients is a time-consuming process but health insurances—as well as hospitals—push more and more to reduce the time spent with patients, as costs of medication, exams, and personnel are dramatically increasing.

Scientists are even farther away from this process, as they often do not have direct contact with the patients. Current criteria for promotion in the medical field still rely heavily on individual research output such as high impact publications, h-index, grants, and invited lectures. There is tremendous pressure and on top of this pressure, there is really no space for a patient-centric view that needs time, patience and dedication. Especially in a system where these activities are not properly recognized and, as a consequence, rewarded. To change this, institutions need to ensure that their tenure and promotions systems are able to evaluate and recognize the contributions investigators conducting translational medicine make. Many institutions are working in this direction and, for instance, signed the Declaration on Research Assessment (DORA). DORA recognizes the need to improve the ways in which the outputs of scholarly research are evaluated. The declaration was developed in 2012 during the Annual Meeting of the American Society for Cell Biology in San Francisco^4^[4]. It is a worldwide initiative covering all scholarly disciplines and all key stakeholders including funders, publishers, professional societies, institutions, and researchers. It is a first step toward assessing research based on its own merits rather than on the basis of the journal in which the research is published.

In conclusion, translational medicine is a very complex branch of medicine. The constant challenges of teaching, researching, publishing, and competing for limited sources of funding, coupled with pursuing career aims and ambitions, can seem daunting. On top of this, we are also adding the patient-centric^4^ view, which adds another level of complexity. However, we believe that once the obstacles are overcome, the real inclusion of patients in the process of translational medicine will improve healthcare delivery to patients.

## Author Contributions

MB conceived the topic, contributed to the topic discussion and wrote the manuscript. PF and NW contributed to the topic discussion and contributed to manuscript writing. FB contributed to the topic discussion and wrote the manuscript.

### Conflict of Interest Statement

The authors declare that the research was conducted in the absence of any commercial or financial relationships that could be construed as a potential conflict of interest.

## Footnotes

^1^https://www.centerwatch.com/drug-information/fda-approved-drugs/drug/100246/lutathera-lutetium-lu-177-dotatate

^2^https://www.parentprojectmd.org/

^3^http://www.jla.nihr.ac.uk/about-the-james-lind-alliance/about-psps.htm

^4^https://sfdora.org/

## References

[1] Cohrs RJ Martin T Ghahramani P Bidaut L Higgins PJ Shahzad A Translational medicine definition by the european society for translational medicine. Eur J Mol Clin Med. (2014) 2:86 10.1016/j.nhtm.2014.12.002

[2] Hwang TJ Carpenter D Lauffenburger JC Wang B Franklin JM Kesselheim AS. Failure of investigational drugs in late-stage clinical development and publication of trial results. JAMA Intern Med. (2016) 176:1826–33. 10.1001/jamainternmed.2016.6008 27723879

[3] Lowenstein PR Castro MG. Uncertainty in the translation of preclinical experiments to clinical trials. Why do most phase III clinical trials fail? Curr Gene Ther. (2009) 9:368–74. 10.2174/156652309789753392 19860651 PMC2864134

[4] London AJ Kimmelman J. Why clinical translation cannot succeed without failure. Elife. (2015) 4:e12844. 10.7554/eLife.12844 26599839 PMC4657068

[5] Truog RD. Patients and doctors–evolution of a relationship. N Engl J Med. (2012) 366:581–5. 10.1056/NEJMp1110848 22335734

[6] Elwyn G Frosch DL Kobrin S. Implementing shared decision-making: consider all the consequences. Implement Sci. (2016) 11:114. 10.1186/s13012-016-0480-9 27502770 PMC4977650

[7] Elwyn G Frosch D Thomson R Joseph-Williams N Lloyd A Kinnersley P. Shared decision making: a model for clinical practice. J Gen Intern Med. (2012) 27:1361–7. 10.1007/s11606-012-2077-6 22618581 PMC3445676

[8] Else H. Radical open-access plan could spell end to journal subscriptions. Nature. (2018) 561:17–8. 10.1038/d41586-018-06178-7 30181639

[9] King KM Pardo Y-J Norris KC Diaz-Romero M Morris D Vassar SD. A Community-academic partnered grant writing series to build infrastructure for partnered research. Clin Transl Sci. (2015) 8:573–8. 10.1111/cts.12327 26365589 PMC5300023

[10] Paberzs A Piechowski P Warrick D Grawi C Choate C Sneed G. Strengthening community involvement in grant review: insights from the Community-University Research Partnership (CURES) pilot review process. Clin Transl Sci. (2014) 7:156–63. 10.1111/cts.12141 24456508 PMC5350939

[11] Schoemaker CG Armbrust W Swart JF Vastert SJ van Loosdregt J Verwoerd A. Dutch juvenile idiopathic arthritis patients, carers and clinicians create a research agenda together following the James Lind Alliance method: a study protocol. Pediatr Rheumatol Online J. (2018) 16:57. 10.1186/s12969-018-0276-3 30219072 PMC6139167

[12] Abma TA Dialogue and deliberation: new approaches to including patients in setting health and healthcare research agendas. Action Res. (2018) 22:147675031875785 10.1177/1476750318757850

